# The Prevalence and Knowledge of Digital Eye Strain Among the Undergraduates in Riyadh, Saudi Arabia

**DOI:** 10.7759/cureus.37081

**Published:** 2023-04-03

**Authors:** Tariq M Almudhaiyan, Tariq Aldebasi, Raghad Alakel, Lujain Marghlani, Abdulrahman Aljebreen, Othillah M Moazin

**Affiliations:** 1 Ophthalmology, Ministry of National Guard - Health Affairs, Riyadh, SAU; 2 Pediatric Ophthalmology, King Abdullah Specialized Children's Hospital (KASCH), Riyadh, SAU; 3 College of Medicine, King Saud bin Abdulaziz University for Health Sciences (KSAU-HS), Riyadh, SAU; 4 College of Medicine, Majmaah University, Riyadh, SAU; 5 Medicine and Surgery, Imam Mohammad Ibn Saud Islamic University, Riyadh, SAU

**Keywords:** dry eye disorder, prevalence, university student, electronic device, computer vision syndrome (cvs), computer vision syndrome

## Abstract

Aim/background

Digital eye strain, also called computer vision syndrome (CVS), is a group of symptoms resulting from prolonged computer, tablet, e-reader, and cell phone use. The level of discomfort and the severity of these symptoms appear to increase with the amount of digital screen use. These symptoms include eyestrain, headaches, blurred vision, and dry eyes. This study aims to assess the changes in the prevalence of digital eye strain among college students in Riyadh, Saudi Arabia.

Methods

A cross-sectional study was conducted among university students at different college institutions in Riyadh, Saudi Arabia. Subjects were interviewed, and the data were collected using an online questionnaire. The questionnaire was composed of student demographic data, students' general knowledge and risk perception of digital eye strain, and the assessment of CVS symptoms questionnaire.

Results

Of the 364 university students, 55.5% were females, and 96.2% were aged between 18 and 29 years. A significant proportion of university students (84.6%) were using digital devices for five hours or more. The proportion of university students who were aware of the 20-20-20 rule was 37.4%. The overall prevalence of positive for CVS symptoms was 76.1%. Independent risk factors for CVS symptoms were gender female, ocular disorders, and using digital devices at a shorter distance.

Conclusion

There was a high prevalence of CVS symptoms among university students in our region. Female students with an ocular disease were more likely to exhibit CVS symptoms than other university students, but using a digital device at a longer distance could alleviate the symptoms of CVS. A longitudinal study is needed to establish the effect of CVS symptoms among university students, especially during the post-pandemic era.

## Introduction

Digital eye strain, also called computer vision syndrome (CVS), is a group of symptoms resulting from prolonged computer, tablet, e-reader, and cell phone use. The level of discomfort and the severity of these symptoms appear to increase with the amount of digital screen use.

These symptoms include eyestrain, headaches, blurred vision, and dry eyes. Many risk factors might contribute to these symptoms; some of these factors are poor lighting, uncorrected refractive errors, glare on the digital screen, and improper viewing distance. One of these factors can cause this syndrome or a combination of these factors [1].

Digital eye strain symptoms can usually be alleviated by obtaining regular eye care and making changes in how the screen is viewed. For example, specific eyeglasses or contact lenses might be prescribed to meet the unique visual demands of computer viewing.

There is a cross-sectional study that was conducted during a period of one year from January 2015 to January 2016 at Al Qassim University, Al Qassim, in Saudi Arabia. This study used a questionnaire to collect relevant data including demographics and various variables to be studied. It shows that a total of 634 students with a mean age of 21 were included as study subjects. Of the total patients, the majority (459, 72%) presented with acute eye symptoms, while the remaining had chronic problems. Thus, this study concludes that continuous use of computers and other electronic devices for long hours is found to have severe problems of vision, especially in those who are using these devices for a long duration [2].

In Riyadh, a study conducted in 2017 among female business and medical students at King Saud University found that there was a higher prevalence of CVS-related symptoms among business students, with headaches being reported by 66% of participants as the most prevalent symptom [3]. A study done on medical students in Riyadh in 2020 found that CVS was highly associated with medical students who use computers and tablets as their primary means of studying, with a prevalence of 70.8% [4].

To determine the prevalence, risk factors, and awareness of CVS, a cross-sectional descriptive study was conducted at King Abdulaziz University, Jeddah. Data was collected through an electronic survey and analyzed using Statistical Package for Social Sciences (SPSS), v21 (IBM Corp., Armonk, NY). A high prevalence of CVS was observed in this study with several (558, 95%) participants noting at least one symptom. Symptoms included excessive tearing and neck, shoulder, and back pain. Female students, students with astigmatism, and students with dry eye disease showed a high association with CVS. However, myopic and hyperopic students showed no association, and spectacles and contact lenses had no association with CVS. Risk factors of CVS in this study were daily long usage, a short distance from the screen, and high brightness. Preventive measures taken were the 20-20-20 rule [5].

A study assessing the prevalence of CVS among health professions students in Jeddah found that CVS was more common in female students than in male students and in students who wear eyeglasses. Moreover, the most reported two symptoms were headache and temporary long- or short-sightedness with a percentage of 68 and 65, respectively [6].

Digital eyestrain is a condition that may affect a student’s academic performance and quality of life, given that virtual learning is becoming normal, and undergraduates spend a lot of their time on devices. It is of great importance that we estimate the prevalence of digital eyestrain after the pandemic to delineate the scope of the problem and whether the current times have changed the prevalence rates as there has been a paucity of recent local studies estimating the prevalence of digital eyestrain among undergraduates.

## Materials and methods

This is a cross-sectional study conducted among university students at different college institutions in Riyadh, Saudi Arabia. After getting the ethical approval from King Abdullah International Medical Research Center (KAIMRC) with IRB approval number: IRB/0666/22, subjects were interviewed, and the data were collected using an online questionnaire. The questionnaire was composed of student demographic data, students' general knowledge and risk perception of digital eye strain, and the assessment of CVS symptoms questionnaire. This study was conducted in Riyadh, Saudi Arabia. Riyadh is the capital of Saudi Arabia and the largest city on the Arabian Peninsula. Also, Riyadh is the second largest city in the Arab world and the 38th largest city in Asia. Its population is 7.6 million people; therefore, Riyadh provides an ideal location for ascertaining the knowledge and the prevalence of digital eye strain among a large number of college students from different specialties in Riyadh, Saudi Arabia. Our inclusion criteria were college students from different specialties in Riyadh, Saudi Arabia.

**Figure 1:**
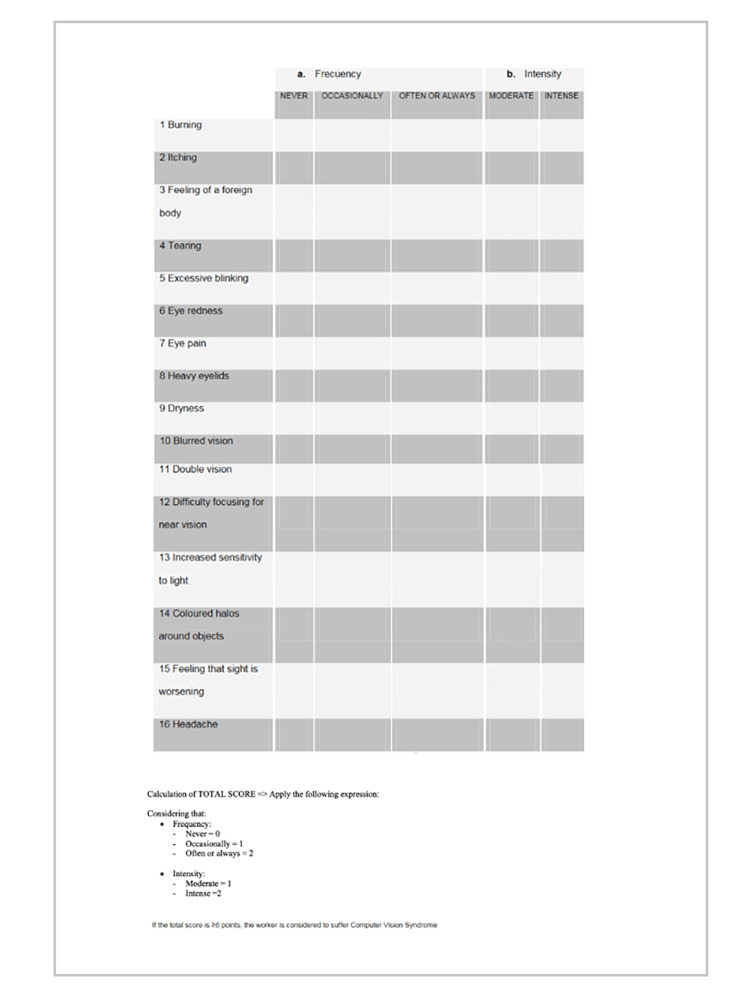
Computer Vision Syndrome Questionnaire (CVS-Q)

The main goal of the questionnaire was to find out the prevalence of CVS using the frequency and intensity of the symptoms experienced by the participants based on the Computer Vision Syndrome Questionnaire (CVS-Q) in (Figure 1).

The frequency was examined according to the 16 components of the questionnaire: never, which means had no symptom at all and is given a score of 0; occasionally, which means intermittent or once a week and is given a score of 1; and often, which means at least twice a week and is given a score of 2. The intensity was evaluated according to two categories: moderate is given a score of 1; intense is given a score of 2. Then, for each symptom, the frequency score was multiplied by the intensity score, and the result was re-coded as follows: 0 = 0; 1 or 2 = 1; 4 = 2. Finally, the recorded result for each of the 16 symptoms was summed up to give a total score. A total score of ≥6 was classified as a CVS-positive symptom.

Both descriptive and inferential statistics were generated using the Statistical Package for Social Sciences (SPSS), version 26 (IBM Corp., Armonk, NY). For categorical data, frequencies and percentages were used to calculate descriptive statistics, whereas means and standard deviations were calculated for continuous variables. The relationship between CVS symptoms according to the sociodemographic characteristics and the related practices of university students toward video display terminal devices has been evaluated using the Chi-square test. A multivariate regression model was performed based on significant results to determine the independent factors associated with positive CVS symptoms with corresponding odds ratios and 95% confidence intervals. A p-value of less than 0.05 was taken as statistically significant. All data analyses were carried out using the SPSS, version 26.

## Results

This study involved 364 university students. As described in Table 1, the most common age group was 18-29 years, with more than half being females (55.5%). Nearly 60% of the students were enrolled in the medical field. The most commonly diagnosed ocular disease was nearsightedness (28.3%).

**Table 1:** Sociodemographic characteristics of the university students (n = 364)

Study Data	N (%)
Age group	
18–29 years	350 (96.2%)
30–39 years	08 (02.2%)
40–49 years	05 (01.4%)
≥50 years	01 (0.30%)
Gender	
Male	162 (44.5%)
Female	202 (55.5%)
Major	
Medical field	215 (59.1%)
Non-medical field	149 (40.9%)
Having ocular disease	
None	118 (32.4%)
Myopia	103 (28.3%)
Hyperopia	39 (10.7%)
Aberration	35 (09.6%)
Astigmatism	69 (19.0%)

**Figure 2:**
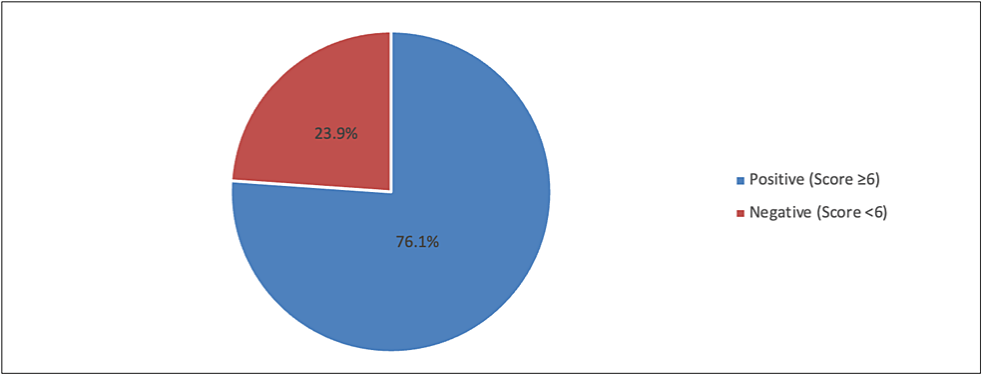
Prevalence of computer vision syndrome using CVS-Q16 CVS-Q: Computer Vision Syndrome Questionnaire.

In Figure 2, the prevalence of students who were positive for CVS was 76.1%, and the rest were negative (23.9%).

In Table 2, the most common study method was the electronic method (65.9%). About 31.9% indicated that their university uses virtual learning very frequently. Nearly all (84.6%) were using electronic devices for five hours or more, with 36.1% taking a break every hour. Among them, 36.1% expressed taking breaks for more than 15 minutes. Approximately 56.3% indicated that the distance between the eye and the screen was less than 40 centimeters, and the seating position was mainly bent back (53%). Also, 47% and 34.3% preferred a dull monitor and a dull illuminated room when using electronic devices. The prevalence of students who were aware of the 20-20-20 rule was 37.4%.

**Table 2:** Assessment of practice and the environment of electronic device use (n = 364)

Statement	N (%)
What is your most utilized method of studying?	
Mostly hard copy	30 (08.2%)
Mostly electronic methods	240 (65.9%)
Both equally	94 (25.8%)
How frequently does your college use virtual learning (online-based academic activities/lectures)?	
Not frequently (<2 h per week)	127 (34.9%)
Slightly frequent (2-9 h per week)	121 (33.2%)
Very frequent (≥10 h per week)	116 (31.9%)
How many hours do you spend per day on electronic devices?	
<2 hours	03 (0.80%)
2-4 hours	53 (14.6%)
5 hours or more	308 (84.6%)
Do you take breaks during the use of electronic devices?	
Yes	183 (50.3%)
No	181 (49.7%)
How often do you take breaks during the use of an electronic device? (n = 183)	
Every 30 minutes	56 (30.6%)
Every hour	66 (36.1%)
More than every hour	61 (33.3%)
What is the average duration of your breaks? (n = 183)	
<5 minutes	20 (10.9%)
5-10 minutes	59 (32.2%)
11-15 minutes	38 (20.8%)
More than 15 minutes	66 (36.1%)
While using electronic devices, the distance between my eye and the screen is approximately	
Less than 40 cm (less than an arm’s length away)	205 (56.3%)
Between 40 and 76 cm (about an arm’s length away)	103 (28.3%)
More than 76 cm (more than an arm’s length away)	07 (01.9%)
I don’t know	49 (13.5%)
While using electronic devices, most of the time my seating position is	
Upright with a straight back	73 (06.9%)
Bending my back	193 (53.0%)
Lying down	98 (26.9%)
How bright is your monitor?	
Very bright	25 (06.9%)
Bright	142 (39.0%)
Dull	171 (47.0%)
Very dull	26 (07.1%)
How well-illuminated is the room during your usage of electronic devices?	
Very bright	115 (31.6%)
Bright	102 (28.0%)
Dull	125 (34.3%)
Dark	22 (06.0%)
Are you aware of the 20-20-20 rule? (Every 20 min, look at an object 20 ft away for 20 s)	
Yes	136 (37.4%)
No	228 (62.6%)

In univariate analysis (Table 3), it was observed that the prevalence of students who had CVS symptoms was significantly more common among females (p < 0.001), those who had an ocular disease (p < 0.001), those who were using virtual learning more frequently (p = 0.017), those who were using electronic devices in the shorter distance (p < 0.001), and those who bent their back when using electronic devices (p = 0.004).

**Table 3:** Relationship between the CVS according to the sociodemographic characteristics and the related practices of university students toward video display terminal devices (n = 364) ^§^p-value has been calculated using the Chi-square test. **Significant at p < 0.05 level. CVS: Computer vision syndrome.

Factor	CVS symptoms		P-value^§^
	Positive N (%) (n = 277)	Negative N (%) (n = 87)	
Gender			
Male	105 (37.9%)	57 (65.5%)	<0.001**
Female	172 (62.1%)	30 (34.5%)	
Major			
Medical field	158 (57.0%)	57 (65.5%)	0.161
Non-medical field	119 (43.0%)	30 (34.5%)	
Having ocular disease			
No	70 (25.3%)	48 (55.2%)	<0.001**
Yes	207 (74.7%)	39 (44.8%)	
Mostly utilized method of studying			
Mostly hard copy	27 (09.7%)	03 (03.4%)	0.073
Mostly electronic methods	175 (63.2%)	65 (74.7%)	
Both equally	75 (27.1%)	19 (21.8%)	
Frequency of institutional virtual learning			
Not frequently (<2 h per week)	86 (31.0%)	41 (47.1%)	0.017**
Slightly frequent (2-9 h per week)	95 (34.3%)	26 (29.9%)	
Very frequent (≥10 h per week)	96 (34.7%)	20 (23.0%)	
Number of hours spent on electronic devices per day			
<5 hours	40 (14.4%)	16 (18.4%)	0.373
≥5 hours	237 (85.6%)	71 (81.6%)	
Approximate distance between the eye and the screen			
<40 cm	169 (70.4%)	36 (48.0%)	<0.001**
≥40 cm	71 (29.6%)	39 (52.0%)	
Seating position when using electronic devices			
Upright with a straight back	45 (16.2%)	28 (32.2%)	0.004**
Bending my back	151 (54.5%)	42 (48.3%)	
Lying down	81 (29.2%)	17 (19.5%)	
Brightness of monitor			
Very bright/Bright	131 (47.3%)	36 (41.4%)	0.334
Very dull/Dull	146 (52.7%)	51 (58.6%)	
Illumination of the room during usage of electronic devices			
Very bright/Bright	161 (58.1%)	56 (64.4%)	0.300
Dull/Dark	116 (41.9%)	31 (35.6%)	
Aware of the 20-20-20 rule			
No	176 (63.5%)	52 (59.8%)	0.526
Yes	101 (36.5%)	35 (40.2%)	

When conducting a multivariate regression model (Table 4), it was found that female gender and ocular disease were the significant independent predictors of increased risk for positive CVS symptoms, while using digital devices for long distances was the significant independent predictor of decreased risk for positive CVS symptoms. This further suggests that compared to male students, female students were predicted to increase the risk of having CVS symptoms by at least 2.27 times (adjusted odds ratios [AOR] = 2.275; 95% CI = 1.248-4.147; p = 0.007). Students with refractive errors were predicted to increase the risk of having CVS symptoms by at least 3.13-fold higher as compared to those who do not have a visual disorder (AOR = 3.131; 95% CI = 1.763-5.562; p < 0.001). In contrast, compared to students who were using electronic devices at a shorter distance, students who were using electronic devices at a longer distance were predicted to decrease the risk of CVS symptoms by at least 54% (AOR = 0.458; 95% CI = 0.255-0.821; p = 0.009). However, the frequency of institutional virtual learning and preferred seating positions when using electronic devices had no significant effect on the CVS symptoms after adjustment to a regression model (p > 0.05).

**Table 4:** Multivariate regression analysis to determine the significant independent factor associated with positive CVS symptoms (n = 364) **Significant at p < 0.05 level. Ref means reference; when you compare each category, compare them with the ref category. AOR: Adjusted odds ratio; CI: Confidence interval; CVS: Computer vision syndrome.

Factor	AOR	95% CI	P-value
Gender			
Male	Ref		
Female	2.275	1.248–4.147	0.007**
Having ocular disease			
No	Ref		
Yes	3.131	1.763–5.562	<0.001**
Frequency of institutional virtual learning			
Not frequently (<2 h per week)	Ref		
Slightly frequent (2-9 h per week)	1.732	0.856–3.501	0.126
Very frequent (≥10 h per week)	0.985	0.460–2.107	0.968
Approximate distance between the eye and the screen			
<40 cm	Ref		
≥40 cm	0.458	0.255–0.821	0.009**
Seating position when using electronic devices			
Upright with a straight back	Ref		
Bending my back	1.835	0.798–4.220	0.153
Lying down	1.251	0.611–2.565	0.540

## Discussion

This study investigated the prevalence of digital eye strain (DES) among university students in Riyadh, Saudi Arabia. The prevalence of DES was measured by utilizing the Computer Vision Syndrome Questionnaire (CVS-Q), which includes 16 symptoms related to CVS; a severity score of 6 or more indicates CVS positive [7]. The findings of this study revealed that the prevalence of CVS symptoms among university students was 76.1%. This is consistent with the study carried out among students at Qassim University, with a prevalence of 72% [2]. This concurred with the study of Alamro et al., wherein 69.8% of female university students exhibited CVS symptoms [4]. However, among school children aged between six and 18 years, the prevalence was lower than the university students, with 35.4% [8]. Similarly, among Indian school children studying online, the prevalence of DES was 50.2%, with 26.3% in the mild degree, 12.9% in the moderate grade, and 11.1% in the severe degree. The COVID-19 pandemic plays a big factor in the high prevalence of DES among students [9]. Therefore, constant monitoring and awareness programs to regulate our students about the hazardous effect of the excessive usage of digital devices are necessitated.

Data from this study suggest that female students and those diagnosed with the ocular disorder were at higher risk for CVS symptoms. These findings mirrored the study of Abudawood et al. According to their reports, female students had a higher chance of CVS. Furthermore, they noted that students diagnosed with astigmatism were associated with CVS. This could be because the eye is not able to focus light evenly on the retina, resulting in distorted or blurry vision that forces the eyes to strain to see more clearly. However, no association was observed in their study among those with myopia or hyperopia [5]. Likewise, Altalhi et al. found an association between CVS symptoms and female gender, but the number of hours spent on a digital device (DD) showed no greater impact on the eye symptoms [6]. In Thailand, based on multiple regression analysis, they found that age (≤15 years), overall DD usage (>6 hours) per day, online learning (>5 hours) per day, multiple DD usage, refractive errors, presence of back pain, and presence of neck pain were determined as significant predictors of CVS [10]. In our study, no difference was observed between CVS symptoms in terms of the student's major, the most utilized method of studying, duration of DD use, the brightness of the monitor, and the room.

According to our results, the top five most debilitating symptoms of CVS were dryness (29.7%), headache (26.9%), worsening sight (26.6%), burning (17%), and itching (16.5%), while double vision represented the least symptom. Studies suggest that headache was the most prominent symptom complained by the students [3-4,6,8,9,11,12]. Contradicting these reports, Abudawood et al. revealed that excessive tearing was the students' most common symptom, followed by shoulder and back pain [5]. The study of Gammoh echoed this as 59% of the physiotherapist students complained of teary eyes, followed by headaches (53%) and increased sensitivity to light (51%), while double vision was the least reported (18.3%) [13].

Excessive use of DD is a detrimental factor for CVS. Our subjects were not different from this scenario, although some of them were unavoidable since they are related to online studying. However, the reality of suffering from various CVS symptoms could be attributed to this habit. Most of these contributing factors were the pattern of virtual learning. For instance, the current studying methods are mostly electronic (65.9%), and virtual learning was conducted more frequently than pre-pandemic era (31.9%). Most students (84.6%) used electronic devices (ED) for more than five hours daily. Only half of our subjects (50.3%) reported taking breaks when using ED. This observation may have been in accordance with the study of Alamro et al. Based on their reports, the electronic form of studying steered to an increased incidence of CVS, and female students were at higher risk than their male counterparts [4]. An increased spending time using DD was seen among Thailander students. More than 60% of the students use DD for at least seven hours/day, and many of them (40%) use various DD during virtual learning [10]. However, in a study by Mohan et al., comparing the use of DD before and during the COVID-19 pandemic, it was discovered that only 1.8% were using DD for more than five hours per day before the pandemic. However, 36.9% of schoolchildren during the pandemic used DD for the same duration. The author emphasized the role of the parents in monitoring the use of DD among their children [9].

It is important to note that our subjects did not widely practice preventive factors for CVS. For example, the majority of our students (56.3%) were using ED for a closer range (<40 cm distance from the eye), while 53% preferred bent back when using ED at a seating position. Among students in Jeddah, the most commonly applied preventive practice was altering display brightness according to the surrounding light brightness (82%). Other preventive factors were practiced less, including taking breaks while using the device (66%), sitting with the screen at face level (59%), sitting with the top of the screen at eye level (43%), sitting with the screen more than 50 cm away (32%), and using antiglare filter (16%) that was also consistent with our reports [6]. In another study done in Jeddah, the most important preventive measure to mitigate the symptoms was applying the 20-20-20 rule, which is for every 20 minutes spent using a screen, the person should look at something 20 feet away for 20 seconds [5]. However, in our study, only 37.4% practiced the same rule, which was not a significant factor for CVS symptoms (p = 0.526).

Limitations

The findings of this were subjected to some study limitations. First, the age of the subjects was not collected appropriately by groups. Thus, we cannot measure if age is a factor in CVS. Also, an online survey is prone to answering bias that may not be a true representative of factual basis. Second, the subjects have not been examined physically, and there has been no test for ocular dryness to correlate with the questionnaire. Thus, the validity of the questionnaire is questionable. Finally, being cross-sectional is prone to disadvantages, including cause-and-effect relationships and bias.

## Conclusions

The prevalence of DES among university students was 76.1%. CVS was widely prevalent in female students and those with ocular disease. However, our students agreed that a longer distance of the DD from the eye is a mitigating factor for CVS. In addition, the COVID-19 pandemic drove many learners to virtual studying. Hence, the trend of CVS symptoms is increasing. Therefore, awareness campaigns are necessary to educate students about safe DD use. Practicing the 20-20-20 rule should be promoted to decrease the prevalence of CVS symptoms among university students.
